# Modern Sociology

**Published:** 1900-06-16

**Authors:** 


### Modern Sociology.

### YOUNG CRIMINALS.

At the beginning of a paper on the subject of juvenile offenders, read before the Royal Statistical Society by Miss Rosa M. Barrett, the author places a French motto, which may be thus translated, "It is easy enough to imprison a man, but it is difficult to release him." It is this fact which makes it a problem how to deal with criminals. It would be both cruel and absurd to condemn a man to imprisonment for life for every offence, however trivial, and yet the difficulty remains, how to send him back to society a better, and not a worse, man than he was before his punishment. In the case of adults, it is almost impossible; and the best hope of reforming the criminal lies in catching him before his evil habits are fully formed, and putting him in the way of leading a nobler life. Therefore the thoughts of all reformers are turned to the juvenile offender, the child who, through evil example and bad environment, has committed an offence, the seriousness of which he himself perhaps does not know. It would be better still if the child could be taken hold of before he has committed any offence; but in those circumstances there is no excuse for society undertaking the charge of him, to the exclusion of his parents, whose natural duty it is.

Child criminals are, unfortunately, common enough. In 1896, more than 1,400 children under 16 years of age were committed to prison in England. A third of the burglars convicted were under 21, and a fourth of all those convicted of larceny were under 16. There were even so many as 60 children under 12 years of age punished by imprisonment. Are these young lives lost to honour, right, and social duty because they have once committed a crime? Parents in all classes know that children are inclined to steal, just because they do not realise the meaning and the rights of property; and if this tendency is to be found in children who are well-trained, and who lack no necessary or comfort of life, is it wonderful that it should be found in those whose circumstances are less fortunate? And yet a crime is a crime. It must not be condoned or excused, or the offender is made worse. The point is to give punishment in such a way that it shall be corrective and not, even in seeming, vindictive.

It was in 1837 that special consideration began to be given to youthful criminals. Inquiries made at that date showed that there were children of seven in the common prisons, and boys of 10 in the hulks and convict-ships. They were allowed much freer companionship with adult offenders than would be permitted now; they lost the salutary fear of the gaol; they were on their way to become habitual criminals. Some private efforts had been made to differentiate between juvenile and adult offenders, but the first official attempt was made in 1838, when Parkhurst was established as a school for criminals from 16 to 18 years of age. The methods of this institution were, however, too much akin to those of the prison, and the place was closed in 1864. Whipping was first administered to juvenile offenders in 1847, the age-limit being 14; in 1861 the age was raised to 16. At present about 3,000 boys are whipped annually, always under careful regulation. Since 1874, nearly all juvenile cases can be dealt with by a magistrate, and thus the necessity for keeping the accused under arrest until assizes is done away with. Moreover, any offences are now punishable by fine instead of by imprisonment; and since 1879, under the First Offenders' Act, a judge may release the young delinquent on his own recognisances to come up for judgment if called upon. The inclination here is to let the young offender go, and give him another chance; but there is a lack of the machinery, employed in other countries, for keeping him under observation until he has proved himself inclined to well-doing, nor is there any machinery for keeping him under probation. There are in this country no reformatories, such as that at Elmira, for first offenders over 16. After that age the delinquent must go to prison. And it is admitted by almost everyone that gaols make gaol-birds. But this knowledge makes judges and magistrates unwilling to send a youth to prison. Thus it comes about that juvenile prisoners are decreasing in numbers throughout the United Kingdom, while the number of criminals between the age of 16 and 21 is increasing. There is, however, a fairly steady decrease in the number of offenders under 16. The number of juvenile offenders committed to prison in 1896 was, in England and Wales, 1,498. This is the lowest number on record, and compares satisfactorily with the 13,981 who were committed in 1856. In Scotland, in 1896, the total number was 618, and in Ireland 210. To these, however, should be added the number admitted into reformatories, viz., 1,170. The admission to reformatories does not vary much from year to year, although for a few years in the eighties the number of children sent to these institutions rose to over 6,000. There is, on the other hand, a steady increase in the numbers admitted into industrial schools. In 1864, when they were first opened, 1,668 were sent to them, while in 1896 the admissions numbered 6,251 boys and 1,083 girls.

As for the antecedents of the young people who are to be found in our prisons, it may be said that nearly all belong, if not to the absolutely criminal, at least to the disreputable classes. It is reckoned that at least a fifth of the juvenile criminals in England are the offspring of drunken parents. Moreover, it is found that criminals generally are uneducated. Of the number of convicted prisoners in England in 1896 only 3,167 could read and write well, 31,221 not at all, and 114,460 very little. Moreover, the criminal is very rarely a skilled workman. The great majority—80,000 out of 100,000—have no definite occupation, and whatever honest employment they may have is of a casual and precarious nature. Education, then, both general and technical, is the best preventive of crime. Akin to this is insistence on the steady attendance at the educational opportunities which are now within the reach of all. "Truancy," said Mr. Drew, a member of the London School Board, "is to be credited with nearly all juvenile crime." In Manchester 67 per cent. of the children committed to industrial schools had been street hawkers, and in Leeds 60 per cent. The restless habits engendered by such a mode of life lead readily to crime, and when to this is added the lack of education to enable the truant to compete with his better trained neighbours in maturer life, the road to evil is found ready made.

